# Improving Fibrin Hydrogels' Mechanical Properties, through Addition of Silica or Chitosan-Silica Materials, for Potential Application as Wound Dressings

**DOI:** 10.1155/2021/9933331

**Published:** 2021-06-02

**Authors:** Natalia Y. Becerra, Luz M. Restrepo, Yessika Galeano, Ana C. Tobón, Luis F. Turizo, Monica Mesa

**Affiliations:** ^1^Tissue Engineering and Cell Therapy Group, University of Antioquia, Medellin 050010, Colombia; ^2^Materials Science Group, Institute of Chemistry, University of Antioquia, Medellin 050010, Colombia

## Abstract

Fibrin is a protein-based hydrogel formed during blood coagulation. It can also be produced *in vitro* from human blood plasma, and it is capable of resisting high deformations. However, after each deformation process, it loses high amounts of water, which subsequently makes it mechanically unstable and, finally, difficult to manipulate. The objective of this work was to overcome the *in vitro* fibrin mechanical instability. The strategy consists of adding silica or chitosan-silica materials and comparing how the different materials electrokinetic-surface properties affect the achieved improvement. The siliceous materials electrostatic and steric stabilization mechanisms, together with plasma protein adsorption on their surfaces, were corroborated by DLS and *ζ*-potential measurements before fibrin gelling. These properties avoid phase separation, favoring homogeneous incorporation of the solid into the forming fibrin network. Young's modulus of modified fibrin hydrogels was evaluated by AFM to quantitatively measure stiffness. It increased 2.5 times with the addition of 4 mg/mL silica. A similar improvement was achieved with only 0.7 mg/mL chitosan-silica, which highlighted the contribution of hydrophilic chitosan chains to fibrinogen crosslinking. Moreover, these chains avoided the fibroblast growth inhibition onto modified fibrin hydrogels 3D culture observed with silica. In conclusion, 0.7 mg/mL chitosan-silica improved the mechanical stability of fibrin hydrogels with low risks of cytotoxicity. This easy-to-manipulate modified fibrin hydrogel makes it suitable as a wound dressing biomaterial.

## 1. Introduction

Obtention of biomaterials from natural polymers that can be used in the wound healing process is an important field of study. It could be an efficient and cost-effective way to reduce morbidity in wound care around the world, especially in developing countries [1, 2]. In addition, skin wound care is becoming more relevant due to the problems related to the widespread use of personal protective implements in the global fight against COVID-19 [3]. Different strategies have been explored to improve skin wound treatments by using functional skin grafts made from natural hydrogels with cells [4, 5].

Fibrin is a biopolymer of interest in tissue engineering for building grafts that can be used in wound healing. It is formed during a blood coagulation cascade. Its primary function in wound healing is hemostasis. It is also a provisional extracellular matrix (ECM) cell support for tissue repair through extracellular binding sites [6]. Functional grafts for wound healing can be obtained by modifying hydrogels with cells [7–9], biomolecules such as antioxidants, antimicrobial acids, and peptides [10–12] among others.

Fibrin, as the main component in blood clots, is highly extensible. Consequently, it tends to stretch instead of break. This behavior is highly dependent on the fibrin fibers thickness [6]. Mechanical properties, such as elasticity, are key factors in the stability of blood clots in wounds. Besides, fibrin clots are highly porous networks due to their extremely low protein content. This is important in nutrient diffusion processes. However, it implies that when fibrin networks are subjected to deformation stress, there is a loss of high amounts of water and the network collapses [13]. This phenomenon is related to fiber structural changes at the molecular level and makes fibrin hydrogels unstable and difficult to manipulate [6, 13, 14].

The addition of particles has been largely used as a reinforcement strategy for polymers such as fibrin hydrogels [15]. In these cases, the homogeneous distribution of the reinforcement material in the polymeric matrix is a key factor for achieving mechanical reinforcement [16]. Silica particles have been shown to be a good choice because they are easily synthesized with different particle sizes and surface areas [17–20]. They can improve the physical, chemical, and biological properties of wound dressings as well as play an active role in wound healing [21]. Even if fibrin reinforcement with silica has not been widely explored, improvement of rheology properties during the polycondensation of silica precursors has been evidenced by changes in the viscoelastic modulus of the fibrin hydrogel [22]. Moreover, silica-fibrin hydrogel preserves the scaffold capacity for cell proliferation [23]. Some authors have shown synergic effects of hybrid chitosan-silica materials on wound healing [24, 25] and improved biofunctionality [10]. Chitosan also improved the hemostatic behavior of mesoporous silica, enhancing the behavior of the fibrin network as a physical barrier to prevent a hemorrhage, in *in vivo* and *in vitro* experiments [26]. Chitosan-fibrin hydrogels increased the elongation and tensile strength properties of fibrin films and sponges [12, 27, 28]. Nevertheless, the effect of hybrid chitosan-silica particles as mechanical reinforcers has not been studied yet. Young's modulus, determined from Atomic Force Microscopy (AFM), is useful for determining the material mechanical properties improvements in terms of stiffness. Some examples include compression analysis of fibrin hydrogels and other biomaterials used as scaffolds [15, 29].

Considering that the hybrid nature of the chitosan-silica confers different electrokinetic properties to the silica surface [18], the question that addressed this work was do fibrin hydrogels modified with silica and chitosan-silica materials improve the hydrogel resistance to collapse by compression (stiffness) than nonmodified ones and if this improvement allows the material to be used as scaffolds for human fibroblasts? The objective was to overcome the fibrin mechanical instability due to water loss under deformation, through the addition of silica or chitosan-silica materials during the production of these hydrogels. The first issue was to ensure the colloidal stability of the siliceous materials/human plasma system, in order to obtain a homogeneous dispersion. *ζ*-potential and Dynamic Light Scattering (DLS) were used as predictive tools to infer the system colloidal stability. These techniques allowed confirmation of the adsorption of plasma proteins onto the surfaces of siliceous material, and its consequences on their aggregation before fibrin gelation was induced by calcium chloride.

Young's modulus of both native and modified fibrin hydrogels (after gelation) was determined by AFM to quantitatively describe their stiffness. Finally, the evaluation of the silica and chitosan-silica materials cytotoxicity on human skin fibroblasts cells was evaluated on plastic and fibrin hydrogels (2D and 3D cultures, respectively). Cytotoxicity evaluation was important because it is a prerequisite to assess if the modified fibrin hydrogels allow the construction of functional grafts for wound dressing applications. The results of this work showed which and how much material (silica or chitosan-silica) is necessary to improve the fibrin stiffness without cytotoxic effects while preserving their ability to be used as human fibroblast growth scaffolds.

## 2. Materials and Methods

### 2.1. Preparation and Characterization of Silica (S) and Chitosan-Silica (CS) Materials

Silica particles (S) were prepared by the Stöber method [17]. In brief, 1.66 mmol tetraethyl orthosilicate (98% TEOS, Merk) was dropwise added to water (Milli-Q grade):ethanol (Baker):ammonium hydroxide (30%, Merk) 3.4 : 100 : 6.7 volume ratio under stirring at 875 rpm. It was left under magnetic stirring of 350 rpm for 24 hours at room temperature in a closed container to avoid volatilization of ammonia. The material was washed by centrifugation/redispersion four times with milli-Q H_2_O.

Chitosan-silica materials were synthesized by a biomimetic silica sol-gel process [18]. In brief, the chitosan (prepared in buffer phosphate from chitosan exhibiting 85% deacetylation degree and molecular weight 190–375 kDa, Merck) was added dropwise to a sodium silicate solution for having 0.02% w/v chitosan at pH 6.0, 7.0, or 8.0, from now on denoted as CS6, CS7, and CS8, respectively. The mixture was left under quiescent conditions for 5 hours at room temperature. The obtained dispersions were washed three times with deionized water by centrifugation and stored as a humified pellet at 4°C.

The aqueous dispersion of all materials, at near-neutral pH and room temperature, was characterized. The particle size was evaluated by Dynamic Light Scattering (DLS) (Horiba LB 550, 5 scans per sample) and the particle surface charge was measured by *ζ*-potential (Malvern Zetasizer, 3 scans per sample). Synthesis of all materials was carried out four times (*n* = 4 replicates).

### 2.2. Colloidal Stability of Siliceous Materials on the Precursor of Fibrin Hydrogels

The precursor of the fibrin hydrogel was prepared by mixing human plasma (67% v/v), normal saline (18% v/v), and tranexamic acid (1.4%, antifibrinolytic) in water. Then, different materials at different concentrations (S at 4 and 20 mg/mL and CS at 0.7, 1.7, and 4.0 mg/mL) were dispersed in the fibrin precursor by sonication (1 min pulse, 50% output, 10 s on, and 5 s off). The suspensions were incubated at 37°C for 30 minutes without inducing fibrin gelation. Afterward, the solid materials were recovered by centrifugation and washed three times with milli-Q water. The aqueous dispersion of all materials, at near-neutral pH and room temperature, was characterized. The particle size was evaluated by Dynamic Light Scattering (DLS) (Horiba LB 550, 5 scans per sample). The frequency (%) on the *y*-axis of DLS distributions of hydrodynamic size of this manuscript correspond to intensity-weighted distribution, and the polydispersity index (PDI) was calculated as the square of the standard deviation divided by the mean diameter. PDI results are shown in the supplementary material. The particle surface charge was measured by *ζ*-potential (Malvern Zetasizer, 3 scans per sample). Synthesis of all materials was carried out four times (*n* = 4 replicates).

### 2.3. Preparation of Fibrin Hydrogels from Human Plasma Modified with S or CS

The preparation of fibrin hydrogels was performed according to the protocol described by Gaviria et al. [7]. In the process, different concentrations of S (4 and 20 mg/mL) or CS (0.7, 1.7, and 4 mg/mL) materials were mixed with the human plasma in the precursor of the fibrin hydrogel: human plasma (67% v/v), normal saline (18% v/v), and tranexamic acid (1.4%). The sonication was made for a homogeneous suspension (1 min pulse, 50% output, 10 s on, and 5 s off). Fibrin gelation was induced by 1% CaCl_2_ solution (14% v/v), at 37°C for 30 minutes. Hydrogels were formed onto glass coverslips for posterior Atomic Force microscopy (AFM) analysis. The native fibrin hydrogel was prepared in the absence of siliceous materials.

### 2.4. Modified Fibrin Hydrogels' Young's Elastic Modulus Determined by AFM

Young's modulus was determined by AFM (Keysight 5500 AFM) in force spectroscopy mode, using a 5 *µ*m spherical probe. A previously calibrated spherical probe (spring constant: 0.036 nN/nm) was provided by Novoscan®. The same spherical probe was used in all measurements. Multiple force/distance curve measurements were performed over regular 8 × 8 grids forming a map of 30 × 30 µm on the fibrin hydrogel surface. Maps were obtained in at least 6 separated zones of each sample and with 3 sample repetitions at each system (*n* = 3). Samples prepared onto glass coverslips were kept in a saline solution (NaCl 0.9%) at 4°C until measurements were performed. Submersed AFM measurements were performed. For all samples, the maximum force applied was 4 nN, and the curve duration was 1.5 s at a rate of 8 *µ*m/s. Young's modulus values were calculated from Hertz model curve fits, using open-source Java application AtomicJ [30]. Approach curves were used for the analysis, curve portions used were selected at maximum indentations of 300 nm, and a Poisson ratio of 0.5 was selected as fibrin hydrogels behave like biological samples.

### 2.5. Cytotoxicity of Siliceous Materials

Cytotoxicity assays were performed according to the international standard ISO 10993–5:2009. Biological evaluation of medical devices: tests for *in vitro* cytotoxicity: the fibroblasts were grown directly on plastic (2D cultures) and inside fibrin hydrogels (3D cultures). For 2D cultures, cells were placed to adhere for 24 hours. After that, they were exposed to the materials suspensions. For 3D cultures, cells were exposed to the material suspensions during the preparation of the fibrin hydrogels. Cytotoxicity was evaluated by using AlamarBlue for both 2D and 3D cultures after 24 hours and 48 hours of exposure.

Materials suspensions were treated as extracts according to the standard. Positive controls (cells + Phenol), growth controls (GC) (cells + cell culture media), and blank (suspension vehicle not containing the materials nor cells) were used. Growth inhibition or stimulation due to S or CS materials was calculated as the percent difference in reduction between treated and control cells, using the following equation:

(1) $$\begin{array}{c} \text{percent difference in reduction}=\frac{\left(\varepsilon_{OX\lambda 2}*A1\right)-\left(\varepsilon_{OX\lambda 1}*A2\right)}{\left(\varepsilon_{OX\lambda 2}*P1\right)-\left(\varepsilon_{OX\lambda 1}*P2\right)}*100, \end{array}$$

where *ε*_*OXλ*1_=80586L/mol*∗*cm: molar extinction coefficient of AlamarBlue oxidized form at 570 nm, *ε*_*OXλ*2_=117216L/mol*∗*cm: molar extinction coefficient of alamarBlue oxidized form at 600 nm, *A*_1_: observed absorbance reading for the test well at 570 nm, *A*_2_: observed absorbance reading for the test well at 600 nm, *P*_1_: observed absorbance reading for the growth control well (cells + AlamarBlue without materials) at 570 nm, and *P*_2_: observed absorbance reading for the positive control well (cells + AlamarBlue without materials) at 600 nm.

### 2.6. Statistical Analysis

When statistical differences are reported, they are the results of nonparametric tests since they do not assume a specific data distribution. When the analysis was performed on related samples, a paired sample signed test was used for two samples and Friedman ANOVA for three or more samples. Independent multiple samples were analyzed by the Kruskal–Wallis (K–W) test. The significance level for all tests was 0.005. When K–W test results were significant, post hoc tests between pairs of samples were used to determine which pairs show significant differences. Values in the discussion were reported with the following convention: mean ± 2*∗*SD.

## 3. Results and Discussion

Silica (S) and hybrid chitosan-silica (CS) materials were synthesized by the Stöber [17] and biomimetic [18] procedures, respectively. They were characterized by DLS and *ζ*-potential, before and after exposure to the precursor of the fibrin hydrogel (67% human plasma, traces of NaCl and tranexamic acid). These results were the basis for rationalizing the effects of the addition of these materials on the evaluated mechanical characteristics of the fibrin hydrogels and cytotoxicity behavior.

### 3.1. Colloidal Characteristics of S and CS Siliceous Materials in the Fibrin Precursor Medium

Colloidal stability of silica (S) and hybrid chitosan-silica (CS) materials, together with adsorption of protein in human plasma, were studied by DLS and *ζ*-potential.

**Figure 1:**
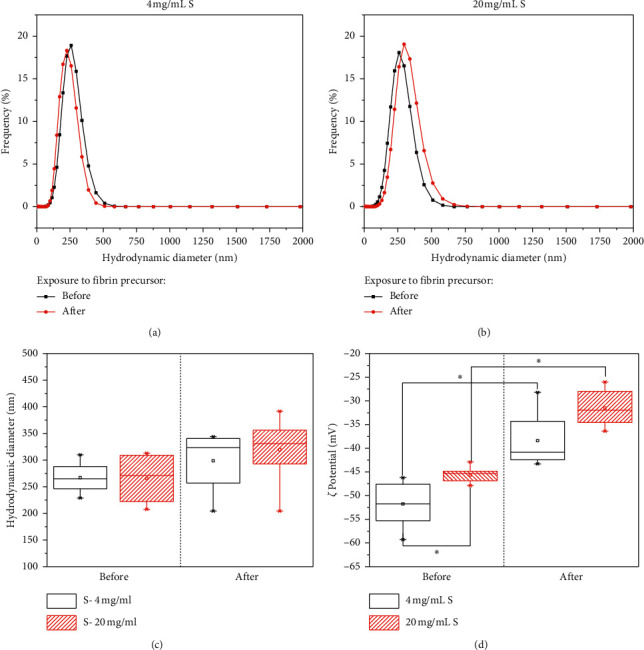
Silica (S) colloidal properties. Hydrodynamic diameter size: distribution for 4 mg/mL S (a) and 20 mg/mL S (b) changes after exposure to the fibrin precursor (c) measured by Dynamic Light Scattering (DLS) and *ζ*-potential results (d).  ^*∗*^Different at a significance level of 0.005 for *n* = 4.

Aqueous suspensions of S material exhibited DLS monomodal hydrodynamic diameter distribution (Figures 1(a) and 1(b)) and negative *ζ*-potential values (Figure 1(d)) at neutral pH. These characteristics are due to the deprotonated silanol groups in the surface, which caused the electrostatic repulsions between particles [31]. The mean size value was around 261.4 ± 70.1 nm independent of the material concentration (Figure 1(c)), with the highest variation for the 20 mg/mL S suspension. The high variation at this concentration is because the probability of particle collisions increases at a higher concentration due to the less negative *ζ* value (Figure 1(d)). However, the *ζ* value is still far from −30 mV, which is the value that predicts the colloidal stability of the S suspension through an electrostatic mechanism [32, 33].

Significant change of the *ζ*-potential was observed for S at all concentrations when the materials were exposed to the precursor of the fibrin hydrogel, washed, and resuspended in water (Figure 1(c)). *ζ*-potential values became less negatives, indicating the possible adsorption of plasma proteins such as fibrinogen, thrombin, and coagulator factors. This adsorption could occur through H-bonds and electrostatic interactions with neutral and deprotonated silanol groups at the surface of the S materials [19]. Salts and tranexamic acid present in the fibrin precursor could also contribute to the *ζ*-potential change; however, they were almost eliminated during washing due to their high solubility in water. The efficient protein adsorption from the hydrogels precursor ensures homogeneous incorporation of the particles in the fibrin network, as has been shown for other polymers and hydrogels [34].

The statistical test for the DLS results did not show a significant difference between the mean hydrodynamic diameter before and after exposure, at each S concentration (Figure 1(c)). Therefore, the protein adsorption did not favor the formation of S material agglomerates. This will be advantageous for effective and homogeneous incorporation of the S materials into the crosslinked fibrin network, without the creation of future fatigue or breakdown points, as has been shown for collagen hydrogels [35]. However, specific care must be placed on the 20 mg/mL S suspension because the *ζ*-potential lied on the colloidal stability limit (Figure 1(d)).

**Figure 2:**
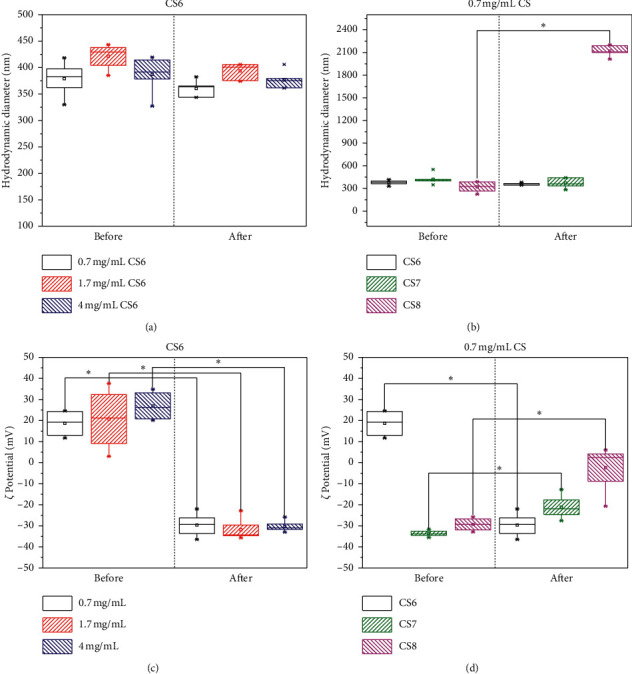
Dynamic Light Scattering (DLS) and *ζ*-potential results for CS materials. Hydrodynamic particle size (*n* = 4 synthesis replicates) and *ζ*-potentials before and after exposure to the fibrin precursor. (a), (c) CS6 at different concentrations and (b), (d) CS6, CS7, and CS8 materials.  ^*∗*^Different at a significance level of 0.005. *n* = 4.

Three chitosan-silica CS materials with different electrokinetic surface properties [18] were used. Their aqueous suspensions at near-neutral pH exhibited similar hydrodynamic sizes and did not vary significantly at different concentrations (Figures 2(a) and 2(b)). On average, they were larger in size and with higher variation (378.6 ± 112.7 nm, Figure 2(a)) than those measured for S aqueous suspensions (Figures 1(a) and 2(b)).

The sign of *ζ*-potential was positive for the CS6 material due to the protonated amine and neutral silanol groups on its surface, independent of the suspension concentration (Figure 2(c)). The other two CS7 and CS8 materials exhibit negative *ζ*-values (Figure 2(d)), following the same tendency reported by Diosa et al. [18]. These differences could be correlated with the chitosan incorporation and protonation state of the free silanol and amine groups of the surface, which in turn affect the surface hydrophilicity. *ζ* values were nearer to zero than those reported for the S materials (Figure 1(c)). However, the presence of chitosan chains, exposed on CS materials surface, can contribute to the colloidal stability in aqueous dispersion precursor through a steric mechanism. This behavior has been seen in silica particles bearing organic groups on their surface [36]. Also, this contribution from the chitosan chains to the material colloidal stability avoided the significant change of hydrodynamic size towards larger values of CS agglomerates in the presence of plasma protein, except for 0.7 mg/mL CS8 suspension (Figures 2(a) and 2(b)). The colloidal behavior of the CS8 sample can be explained by the full-charge charge screening after protein adsorption [19], as corroborated by the mean *ζ*-value around zero (Figure 2(d)). Most likely, the protein-surface affinity could be higher in CS8 than CS7, CS6, and S materials because of the higher chitosan percentage and hydrophilic character, reported by Diosa et al. [10, 18].

On the one hand, these results on the fibrin precursor dispersion indicated that the plasma proteins were adsorbed on S, CS6, and CS7 materials without affecting the colloidal stability. This protein-surface affinity will favor the incorporation of the solid materials into the forming fibrin network. On the other hand, the electrostatic and steric colloidal stabilization will avoid the phase separation (macroscopic precipitates were not seen) and the formation of breakdown points in the fibrin hydrogel.

### 3.2. Mechanical Characteristics of Fibrin Hydrogels Modified with S and CS Materials

**Figure 3:**
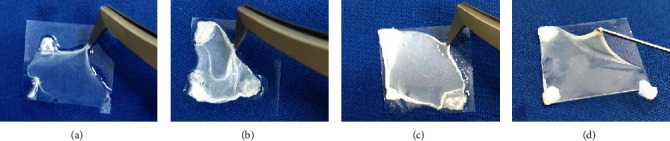
Representative photographs for (a) native and modified fibrin hydrogels at (b) 4 mg/mL S, (c) 20 mg/mL S, and (d) 0.7 mg/mL CS6 materials.

Gelation of the fibrin precursor (human plasma, NaCl isotonic solution, and tranexamic acid) was induced by CaCl_2_. This produced a highly extensive fibrin hydrogel. However, a notable loss of water was observed when it was manipulated (Figure 3(a)). The presence of S or CS materials increased the opalescence of the hydrogels homogeneously, without affecting the extensibility of the hydrogel. Added siliceous materials reduced water loss during the manipulation of the samples (Figures 3(b)–3(d)). This was the first clue that the solid materials were incorporated into the fibrin network, promoted by the interactions with the plasma proteins (discussed in the precedent section). The presence of siliceous materials also improved the facility to manipulate the fibrin hydrogels, especially with CS6 material at 0.7 mg/mL concentration. Easy-to-manipulate behavior shown by modified hydrogels is a positive feature towards using this material as wound dressings because they should be easily applied and removed from the skin.

Additionally, this macroscopic improvement allowed performing AFM force spectroscopy experiments easily; many native nonmodified fibrin hydrogels AFM experiments were lost because of the collapse of the hydrogel. Compression analysis of the stiffness by AFM is useful for describing the mechanical stability of hydrogels and biological samples [37]. Moreover, AFM force spectroscopy applies low compression forces to samples, which is convenient since the loss of water due to compression of fibrin hydrogel can cause instability and network collapse [6].

**Figure 4:**
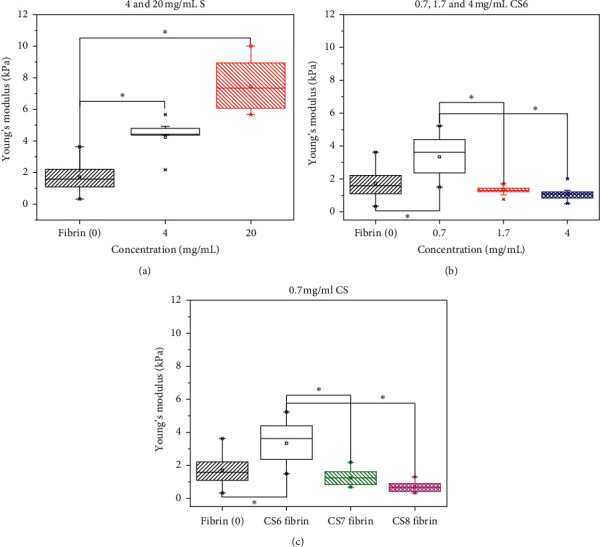
Young's modulus of fibrin hydrogels modified with S and CS materials compared with native fibrin (0). S materials (4 and 20 mg/mL) (a), CS6 materials (0.7, 1.7 and 4 mg/mL) (b), and CS6, CS7, and CS8 materials (0.7 mg/mL) (c).  ^*∗*^Different at a significance level of 0.005. Native fibrin *n* = 9; S and CS modified fibrin *n* = 3.

Mean Young's modulus of nonmodified fibrin hydrogels (1.7 ± 0.28 kPa) determined by AFM was similar to that obtained with low fibrinogen concentration [38]. The presence of 4 and 20 mg/mL S material increased Young's modulus 2 and 3 times, respectively (Figure 4(a)). The affinity of the S material with the fibrinogen promoted its incorporation into the fibrin network. This is because the fibrinogen can be adsorbed on the siliceous surface by electrostatic interaction between the positively charged amino acids (i.e., Lys and Arg) and deprotonated silanol groups at near-neutral pH of the human plasma [39]. This adsorption occurred mainly by the disordered *α*C-chains and fibrinogen strands on the surface [40], which could be beneficial for their polymerization and crosslinking because the presence of particles did not interfere with the fibrin network formation [23]. Electrostatic colloidal stabilization of S materials allowed reaching concentrations up to 20 mg/mL S suspension (Figure 1). However, a high increase of S concentration did not yield a linear increase of Young's modulus as expected. This could be explained because S agglomeration was still probable, as predicted from the low *ζ*-potential of this suspension in the presence of human plasma (Figure 1(c)). Finally, fibrin mechanical reinforcement was more homogeneous with the lowest evaluated S concentration, as indicated by the smaller variation of Young's elastic modulus (Figure 4(a)).

Interestingly, a similar 2-fold modulus increase was achieved with the hydrogel prepared in the presence of 0.7 mg/mL CS6 material (Figure 4(b)) compared to the increase obtained with 4 mg/mL of S. At the first glance, both CS and S are hydrophilic materials that can contribute to the maintenance of fibrin hydrogel hydration, as shown in the literature for other hydrogels [25, 41, 42]. However, the increase of the CS6 concentration was not advantageous for the mechanical reinforcement (Figure 4(b)). Most likely, it was the optimum concentration for the effective chitosan-fibrin(ogen) chains coupling that promoted microstructural changes associated with the improved mechanical properties of fibrin hydrogels, as shown for composites with chitosan and other hydrophilic polymers [15, 27]. Above this concentration, the steric colloidal stabilization could be not enough for avoiding flocculation/agglomeration when the CaCl_2_ was added for inducing the gelling. In contrast to CS6, 0.7 mg/mL CS7 and CS8 materials neither had a significant effect on Young's elastic modulus with respect to the native hydrogel (Figures 4(b) and 4(c)). This fact corroborated the deleterious effect of the particle agglomeration, which was envisaged from the colloidal instability of 0.7 mg/mL CS8 suspension (Figure 2(d)), as a consequence of full-charge screening after protein adsorption [19].

AFM analysis performed on different areas allowed the conclusion that a homogeneous reinforcement was obtained, which agrees with the macroscopic appearance of modified fibrin hydrogels (Figure 3). A similar Young's modulus improvement was achieved when fibrin was modified with 4 mg/mL S and 0.7 mg/mL CS6 materials (Figure 4). These two concentrations were kept constant, to maintain the mechanical properties of the modified fibrin hydrogels with S and CS materials and make other comparisons related with the cytotoxicity towards fibroblasts. The concentration of 4 mg/mL was the lower and upper limit for the silica (S) and chitosan-silica (CS) concentrations, respectively. The exposed chitosan chains in the well-dispersed CS6 fibrin precursor suspension could have improved the growth of fibrin fibers with gelation, which induces a high crosslinking degree, consequently increasing Young's modulus even at a lower concentration. The fact that CS6 material required low concentration to achieve a good fibrin reinforcement could also improve the biological functionality of modified fibrin hydrogels.

### 3.3. S and CS Materials' Cytotoxicity on 2D and Modified Fibrin Hydrogel 3D Human Fibroblast Cultures

The materials used in this study are not evaluated as blood-contacting materials; because of the potential application considered in this work is the reinforcement of fibrin hydrogels for wound dressing, materials will be inside the fibrin networks. For other applications, a hemocompatibility test should be addressed.

**Figure 5:**
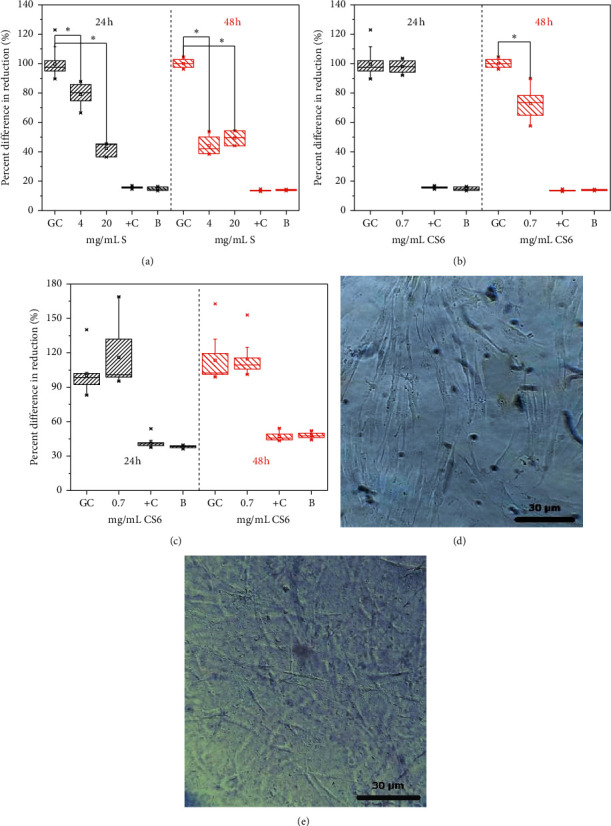
S and CS materials' cytotoxicity on human fibroblasts. 4 and 20 mg/mL S (a) and 0.7 mg/mL CS6 in 2D (b) and 3D (c) human fibroblasts culture. Growth controls: GC, positive controls: +C, and Blanks: B.  ^*∗*^Different at a significance level of 0.005, *n* = 3. (d), (e).

Alamarblue cytotoxicity assays of materials were performed with human fibroblasts (Figure 5(a)), which are one of the main cell types in the skin and, therefore, a key factor in building skin wound dressings [5, 8].

The percent differences in Alamarblue reduction between treated and control cells (growth control), as calculated with equation (1), were around 80% and 40% after 24 hours of exposure to 4 and 20 mg/mL S, respectively. Putting in another way, the growth was inhibited by 20% and 60% with respect to the control cells growth. This indicated a cytotoxic behavior only for the S materials at the 20 mg/mL concentration because growth inhibition was larger than 50%. After 48 hours of exposure, the S materials at both concentrations (4 and 20 mg/ml) were cytotoxic because growth inhibition was around 60%. The measured hydrodynamic size of the S material suspension (Figure 1) was in the same range as those siliceous materials described in the literature as less-cytotoxic particles [43]. However, the concentration of S at 20 mg/mL has been shown to be cytotoxic. This could be explained because increasing the concentration could have also increased the materials bioavailability in the culture, and therefore, membrane cells could have also been damaged by prolonged interactions.

Figures 5(b) and 5(c) show Almarblue assay results for fibroblasts exposed to 0.7 mg/mL CS6 material in 2D and 3D (inside fibrin) cultures. This material at this concentration showed similar fibrin improvement with Young's modulus than S materials. But, in contrast to S materials, CS6 growth inhibition was around 30% in 2D culture after 48 hours, which was half the inhibition observed with S materials after the same amount of time. Similar to the CS6 material adsorption mechanism of proteins from human plasma, evidenced by the markedly *ζ*-potential change after exposure to the fibrin precursor (Figure 2(d)), the CS6 material less-cytotoxic behavior could be related to the efficient adsorption of serum proteins from the cell culture media. Additionally, this behavior agrees with the favored higher adsorption of fibrinogen on positively charged surfaces [44]. The adsorbed protein layer could mediate the interactions between cells and CS6 materials avoiding cellular damage when fibroblast 2D cultures were performed. In conclusion, CS6 colloidal stability through the steric mechanism, after protein adsorption from the cell culture media, can contribute to minimizing the cells'/materials' damaging interactions. On the contrary, negatively charged S materials (according to the *ζ*-potential results, Figure 1(d)), could be less efficient for protein adsorption present in the cell culture media [44] compared to positively charged CS6 materials. This could also increase the materials/cells friction and the consequent damage at a larger concentration.

Cytotoxicity evaluation inside the fibrin hydrogels (3D) corroborates the functionality of CS6 material as a reinforcer to improve fibrin hydrogels' behavior as fibroblast support. Fibroblasts exposed during 48 hours to CS6 materials grew in the same way as the growth controls cells (Figure 5(e)). It seems that, during the first 24 hours of exposure in this 3D culture, the adsorption of proteins from the human plasma of the fibrin precursor on the CS6 material could promote cell proliferation.

After the protein adsorption process produced by the contact between plasma and CS6 materials (evidenced by the markedly *ζ*-potential change after exposure to the fibrin precursor), we were able to produce the 3D cultures: fibrin hydrogels with the CS6 materials and fibroblasts inside by inducing the coagulation with calcium. This adsorbed plasma proteins on the CS materials could act as a “conditioning film” on the CS surface, inducing a better behavior of the fibrin hydrogel, with the CS materials inside, as support for fibroblasts growth and proliferation.

Healthy 2D and 3D cultures were observed after fibroblast exposure to CS6 materials (Figures 5(d) and 5(e)).

## 4. Conclusions

*In vitro* production of fibrin hydrogels from human blood plasma, with improved mechanical properties, was achieved by the addition of 4 mg/mL S silica or 0.7 mg/mL CS6 chitosan-silica materials. There is a correlation between colloidal stability before gelation and both macroscopic properties and stiffness of the fibrin hydrogel. This correlation was used as a basis to rationalize the results.

DLS and *ζ*-potential on the fibrin precursor dispersion of the materials showed that colloidal stability was dependent on the type and concentration of the materials. Stability of S materials, due to electrostatic repulsion, allowed the addition of higher concentration (4–20 mg/mL) than CS materials (0.7–4 mg/mL).

CS6 material, having a lower content of positively charged chitosan chains and hydrophilic surface character, was stable after the adsorption of human plasma proteins by the steric mechanism. Protein-surface affinity promoted the incorporation of siliceous materials into the fibrin hydrogel network. Then, homogeneous modified fibrin hydrogels were obtained, which are stiffer than native ones, making them stable against loss of water during deformation stress. Young's modulus, measured from force spectroscopy AFM mode on randomized areas, was the indicator of the homogeneous stiffness of each sample. It agreed with the macroscopic appearance due to the mentioned colloidal stability of the materials in the fibrin precursor. Young's modulus increased 2.5 times with the addition of 4 mg/mL S silica. A similar improvement was achieved with only 0.7 mg/mL chitosan-silica, which highlighted the contribution of hydrophilic chitosan chains to Young's modulus.

CS6 material was less cytotoxic for human fibroblast growth on 2D and noncytotoxic 3D (fibrin hydrogel) cultures. This is attributed to the organic chains, low concentration, and efficient protein adsorption that mediate nondamaging cell/materials interactions. It even promoted cell proliferation. These results are promising because they add value in the design of reinforced fibrin hydrogels from an autologous source, intended for their use as wound dressings.

## Data Availability

The data used to support the findings of this study are available from the corresponding author upon request.

## Supplementary Materials

Supplementary Materials 1. Polydispersity index (PDI) of S and SC materials before and after contact with human plasma is presented. 2. ATR-FTIR analysis of S and SC materials before and after contact with the human plasma is presented.
