# Supplementary material for: Improving Fibrin Hydrogels' Mechanical Properties, through Addition of Silica or Chitosan-Silica Materials, for Potential Application as Wound Dressings
Source: Int J Biomater. 2021 Jun 2;2021:9933331. doi: 10.1155/2021/9933331 (PMC8192204; doi:10.1155/2021/9933331)
Supplement: Supplementary Materials — 1. Polydispersity index (PDI) of S and SC materials before and after contact with human plasma is presented. 2. ATR-FTIR analysis of S and SC materials before and after contact with the human plasma is presented. [file 9933331.f1.docx]

**Supplementary Material**

**1. Polydispersity index (PDI) of silica (S) and chitosan-silica (CS) materials:** it was calculated as the square of the standard deviation divided by the mean diameter.

The data (Table S1) indicated that samples are highly monodisperse (PDI < 0.1) or moderately polydisperse (PDI between 0.1-0.4), which indicates that no aggregates of particles were formed.

Table S1. Polydispersity Index for DLS intensity weighted distribution results.

| Samples | | Before plasma contact | | | | After plasma contact | | | |
| --- | --- | --- | --- | --- | --- | --- | --- | --- | --- |
|  |  | Mean | SD | PDI | Mean | | SD | PDI |  |
|  |  |  |  |  |  | |  |  |  |
| Silica 4 mg/ml | Replicate 1 | 234.7 | 67.4 | 0.1 | 209.9 | | 59.5 | 0.1 |  |
|  | Replicate 2 | 270.4 | 85.3 | 0.1 | 318.8 | | 97.8 | 0.1 |  |
|  | Replicate 3 | 279.9 | 70.8 | 0.1 | 337 | | 106.4 | 0.1 |  |
|  | Replicate 4 | 309.8 | 78.8 | 0.1 | 351.1 | | 96.3 | 0.1 |  |
|  |  |  |  |  |  | |  |  |  |
| Silica 20 mg/ml | Replicate 1 | 243.1 | 73.5 | 0.1 | 284.5 | | 83.1 | 0.1 |  |
|  | Replicate 2 | 210.9 | 46.9 | 0.0 | 334.5 | | 101.1 | 0.1 |  |
|  | Replicate 3 | 305.1 | 81 | 0.1 | 391.7 | | 105.4 | 0.1 |  |
|  | Replicate 4 | 312.9 | 77.8 | 0.1 | 368.1 | | 91.2 | 0.1 |  |
|  |  |  |  |  |  | |  |  |  |
| CS6 0.67 | Replicate 1 | 418.5 | 141.9 | 0.1 | 382.6 | | 98.5 | 0.1 |  |
|  | Replicate 2 | 371.2 | 101.6 | 0.1 | 343.7 | | 124.1 | 0.1 |  |
|  | Replicate 3 | 394.7 | 128.9 | 0.1 | 365.3 | | 110.1 | 0.1 |  |
|  | Replicate 4 | 397.7 | 123.5 | 0.1 | 361.8 | | 150.4 | 0.2 |  |
|  |  |  |  |  |  | |  |  |  |
| CS6 1.67 | Replicate 1 | 443.6 | 174.4 | 0.2 | 405.9 | | 123.7 | 0.1 |  |
|  | Replicate 2 | 384.9 | 116.9 | 0.1 | 375.3 | | 117.8 | 0.1 |  |
|  | Replicate 3 | 428.8 | 142.6 | 0.1 | 374 | | 117 | 0.1 |  |
|  | Replicate 4 | 404.3 | 137.4 | 0.1 | 400.7 | | 140.4 | 0.1 |  |
|  |  |  |  |  |  | |  |  |  |
| CS6 4 | Replicate 1 | 414.4 | 138.9 | 0.1 | 379.4 | | 135 | 0.1 |  |
|  | Replicate 2 | 327.2 | 117.5 | 0.1 | 374.5 | | 115.2 | 0.1 |  |
|  | Replicate 3 | 419.8 | 126.6 | 0.1 | 376.2 | | 121.3 | 0.1 |  |
|  | Replicate 4 | 388.5 | 112.7 | 0.1 | 361.4 | | 126.1 | 0.1 |  |
|  |  |  |  |  |  | |  |  |  |
| CS7 0.67 | Replicate 1 | 550.9 | 218.6 | 0.2 | 442.9 | | 144.1 | 0.1 |  |
|  | Replicate 2 | 400.8 | 104.6 | 0.1 | 282.3 | | 123.3 | 0.2 |  |
|  | Replicate 3 | 402.4 | 121.1 | 0.1 | 334.5 | | 112.6 | 0.1 |  |
|  | Replicate 4 | 418.6 | 136.2 | 0.1 | 359 | | 123.4 | 0.1 |  |
|  |  |  |  |  |  | |  |  |  |
| CS8 0.67 | Replicate 1 | 392 | 139.1 | 0.1 | 378.6 | | 192.1 | 0.3 |  |
|  | Replicate 2 | 277.5 | 101.2 | 0.1 | 2190 | | 873.8 | 0.2 |  |
|  | Replicate 3 | 223 | 83.3 | 0.1 | 2100.3 | | 649.8 | 0.1 |  |
|  | Replicate 4 | 265.1 | 86.2 | 0.1 | 2011.5 | | 650.2 | 0.1 |  |

**2**. **ATR-FTIR analysis of S and SC materials before and after contact with the human plasma:** spectra were taken for the S and SC materials in a Perkin-Elmer spectrometer (16 scans from 4000 cm^-1^ to 500 cm^-1^, 2 cm^-1^ resolution)

In the case of silica new bands around 1640 cm−1, 1520 cm−1 and 1240 cm−1, corresponding to Amide I, II and III N-H stretching bands of the proteins, corroborate the plasma protein adsorption on this kind of surface (Figure S1). However, the analysis is not straight with chitosan-silica particles. The infrared spectrum of this hybrid material exhibits also the amide signal (Figure S2), due to the chitosan presence with 85% deacetylation (described by Diosa et al [1]). Therefore, the changes on intensity and widening of the bands are related to the protein adsorption and the conformational changes of the human plasma proteins on the solid surface.

Figure S1. ATR-FTIR spectra of S materials before and after contact with human plasma.

Figure S2. ATR-FTIR spectra of CS6 materials before and after contact with human plasma.
